# Giant atypical carcinoid of the liver with vascular metastases and local sinusoidal invasion: a case report

**DOI:** 10.1186/1752-1947-1-47

**Published:** 2007-07-12

**Authors:** Daniel Lingamfelter, Laura Hoffman, Amit Verma, William DePond, Kamani Lankachandra

**Affiliations:** 1 Department of Pathology, University of Missouri-Kansas City School of Medicine and Truman Medical Center, Kansas City, Missouri, USA; 2 University of Missouri-Kansas City School of Medicine and Truman Medical Center, Kansas City, Missouri, USA; 3 Department of Radiology, University of Missouri-Kansas City School of Medicine and Truman Medical Center, Kansas City, Missouri, USA

## Abstract

We present the case of a 46 year old woman with a giant, 23-centimeter, atypical carcinoid of the liver. A primary site for this neoplasm could not be identified despite multiple radiographic imaging studies, including a somatostatin scan, and a thorough inspection of the bowel during surgical resection of the lesion. Histologically, the tumor displayed mild cytologic atypia, abundant necrosis, and intravascular metastases, the last feature of which was identified by immunohistochemical markers for chromogranin and synaptophysin. Also described is the unusual sinusoidal infiltration, or "spillage," of tumor cells into the surrounding liver parenchyma, a feature that has not been described as far as we are aware but may suggest an aggressive clinical course. Even though an exact definition of atypia for these lesions apparently does not exist at this point, the multiple atypical features in this case strongly suggest the diagnosis of atypical carcinoid of the liver, thus far an altogether rare and vaguely reported entity. As more cases arise in the medical literature, it may be worthwhile to establish a set of guidelines to define atypical hepatic carcinoids and other gastrointestinal carcinoids, although survivorship data thus far indicates no significant difference in the prognosis between typical versus atypical variants.

## Background

Primary hepatic carcinoid tumor is an incredibly rare entity but must be distinguished from other lesions such as hepatocellular carcinoma because of its different treatment and prognostic implications. At this time about 125 cases have been reported, but many of these may have been metastases or a neuroendocrine component of another neoplasm [2]. Even rarer is the entity of primary hepatic atypical carcinoid, with only 19 cases so far mentioned in the literature [1]. We present the case of a giant atypical carcinoid tumor that, as far as we can determine, is primary to the liver and displays the unusual histopathologic phenomenon of sinusoidal infiltration throughout the surrounding liver parenchyma.

## Case Presentation

The patient was a 46-year-old white female who presented with vague right upper abdominal pain and fullness for approximately one and a half months' duration. This pain intensified in a seated position. Besides having a chronic history of migraine headaches, she noted that she otherwise felt healthy and had not visited a physician for the past twenty years.

Physical examination revealed a large, firm mass within the right upper quadrant that extended 10 cm below the right costal margin and stretched horizontally from the epigastric midline to the right lateral abdominal wall.

**Figure 1:**
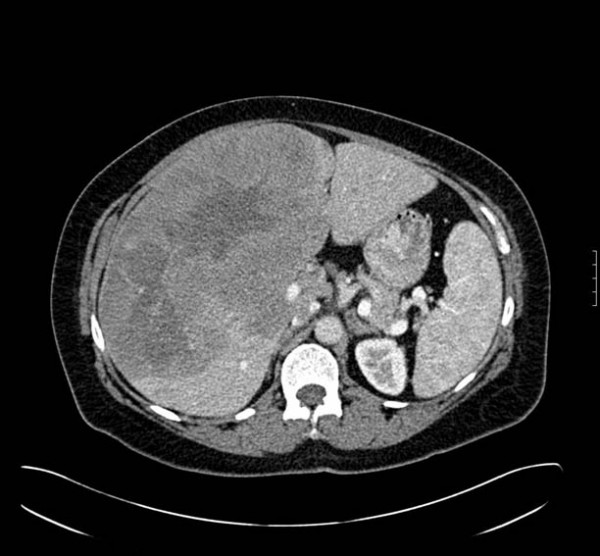
An axial CT image obtained during hepatic arterial phase demonstrates a heterogeneous enhancement of an approximately 22 × 14 cm hepatic mass involving the right hepatic lobe with a central region of hypoattenuation.

An axial CT scan demonstrated a heterogeneous enhancement of a 22 × 14 cm hepatic mass involving the right hepatic lobe with a central region of hypoattenuation (Figure 1). Magnetic resonance imaging showed a 23 × 16 × 14 cm intraparenchymal hepatic mass virtually replacing the right lobe of the liver while medially displacing the portal vein and inferior vena cava. Ingrowth into these vascular structures could not be identified. The right kidney and right hemidiaphragm revealed caudal and cranial displacement, respectively. No other lesions were identified.

An ultrasound-guided liver core biopsy procedure was performed a week later. The pathology report issued the diagnosis "neuroendocrine neoplasm" with a differential diagnosis that included carcinoid tumor, gastrinoma, insulinoma, and hepatocellular carcinoma with neuroendocrine features.

Subsequently, the patient underwent a somatostatin scan, involving the administration of 6 millicuries of indium-111 and use of a plantar gamma camera and single photon emission computed tomography (SPECT). The study identified an abundance of heterogenous activity within the hepatic mass but detected no extrahepatic foci of activity.

Based upon the pathologic and radiologic findings, the surgical team decided to perform a right hepatic lobectomy.

## Materials and methods

5-um sections from formalin-fixed, paraffin-embedded tissue were used for routine light microscopic study and immunohistochemical analysis including the antibodies specified in Table 1. These immunohistochemical stains were performed with a labeled avidin-biotin complex immunoperoxidase method using commercially available monoclonal antibodies and DAB as the chromogen. In order to provide negative controls on patient tissue and thereby ensure specificity of the reactions, the aforementioned antibodies were substituted for an unrelated antibody during the incubation procedure. Formalin-fixed and paraffin-embedded pancreatic tissue was used as a positive control for synaptophysin, chromogranin, and neuron-specific enolase (NSE); hepatic tissue for alpha-fetoprotein (AFP) and Hep-Par1; and epidermis for cytokeratin.

**Table 1:** Immunohistochemical stains used to establish the diagnosis of the neoplasm, including the vendors as well as the clones and dilutions used

Antibody Dilution	Vendor	Clone
Synaptophysin 1:100	Dakocytomation	--
Chromogranin-A 1:100	Dakocytomation	DAK-A3
NSE 1:100	Dakocytomation	BBS/NC/VI-H14
Cytokeratin 1:400	Biocare Medical	AE1/AE3+5D3
Hep-Par1 1:100	Dakocytomation	OCH1E5
AFP Pre-dil	Zymed Laboratories	ZSA06

NSE: Neuron-specific enolase, AFP: alpha-fetoprotein, Pre-dil: pre-diluted.

### Pathologic findings

**Figure 2:**
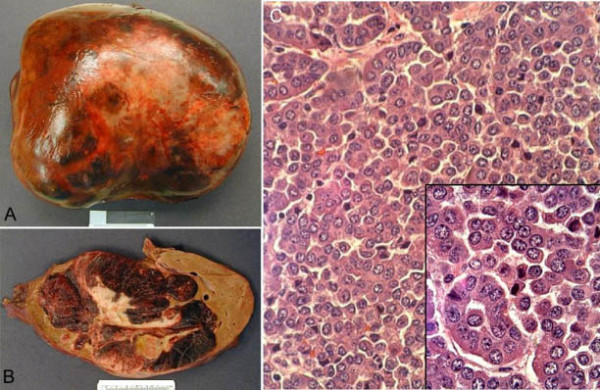
(A) The enormous tumor has all but replaced and distorted the right hepatic lobectomy specimen. (B) Cut sections reveal diffuse areas of necrosis and a large, irregular central scar. (C) The tumor is composed mostly of a trabecular architecture but with some scattered acinar structures (H&E, 400×). The cells show mild atypia while displaying eccentric, "salt and pepper" nuclei and gritty pink cytoplasm (inset, H&E, 1000×).

The 4300-gram right lobe partial hepatectomy specimen revealed a 23 × 17 × 14 cm dark brown-to-tan, multilobulated mass with abundant areas of hemorrhage and necrosis that nearly replaced the entire normal liver tissue (Figure 2A). A large, irregular area of scarring filled the central portion of the neoplasm (Figure 2B). Multiple areas overlying the anterior capsule were pale and indurated, suspicious for capsular involvement by the lesion. Extensive sampling was performed from all areas of the specimen.

The lesion was composed of a monomorphic cell population arranged in a predominately trabecular architecture and with some scattered acinar arrangements (Figure 2C). The borders of the lesion pushed into the surrounding normal liver parenchyma. Cytologically, the cells showed minimal atypia and possessed eccentric, speckled nuclei with cytoplasmic granularity and eosinophilia (Figure 2C, inset). Mitoses were rare while hemorrhage and necrosis were diffusely present in varying degrees. Sections taken from the central area of the tumor showed early scar formation with scattered tumor islands. Capsular invasion was not identified.

**Figure 3:**
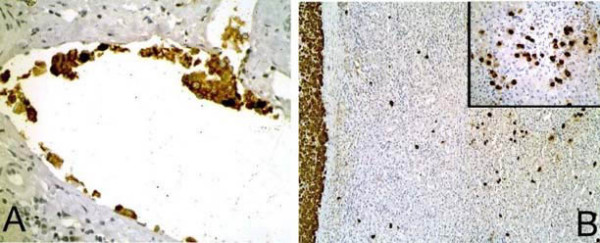
(A) Multiple areas of intravascular invasion are found throughout the liver (H&E, 400×). (B) A stain for the neuroendocrine marker chromogranin reveals the tumor border at the left edge of the photomicrograph (H&E, 100×) with multiple scattered neoplastic cells spilling into the surrounding liver parenchyma (inset, H&E, 400×).

Immunohistochemical stains for chromogranin, synaptophysin, and NSE showed a strong, diffuse positivity for the tumor cells. Multiple intrahepatic, intravascular tumor cell metastases were visualized with these markers as well, especially with chromogranin (Figure 3A). Both the synaptophysin and chromogranin markers highlighted tumor cells scattered throughout the sinusoids of the surrounding liver parenchyma (Figures 3B), suggesting local invasion by individual cells and small cell clusters. None of this sinusoidal "spillage" could be identified at the parenchymal surgical resection margin.

The marker for cytokeratin revealed a cytoplasmic, granular staining pattern while markers for Hep-Par1, alpha-fetoprotein (AFP), and mucicarmine did not highlight the neoplastic cells.

## Discussion

Approximately 125 cases of primary hepatic carcinoids have been described within the literature. Atypical primary hepatic carcinoids appear to be much less common, as the 19 cases reported by Soga in 2002 currently stand as the only such designated entities. Females are affected slightly more often than males (1.4:1), and the ages of the patients have ranged from 18 to 84 with an average age of 54 years [1].

The most common presenting symptom is upper abdominal fullness, with or without hepatomegaly. Abdominal pain and discomfort, diarrhea, and weight loss can be invariably encountered as well. Only about 7% of these lesions manifest the carcinoid syndrome [1], the explanation of which in part stems from hepatic enzymatic degradation of neoplastic-derived products initially spilling into the portal circulation rather than the systemic circulation. Our patient exhibited no symptomatology other than abdominal fullness and mild discomfort. Therefore lab studies to detect carcinoid-related substances were not performed.

The cell from which this entity arises has not been proven, but there is evidence to support a derivation from bile duct epithelium [3,4]. Ultrastructural findings include cell clusters with lumina bordered by cells with microvilli and junctional complexes, similar to the epithelial cells that line bile ducts [3]. Roskams et al. showed that during the earliest stages of regeneration, bile duct epithelium displays neuroendocrine features including cytoplasmic, dense core neurosecretory granules and chromogranin-A expression [4].

Grossly, hepatic carcinoids can vary widely in size, ranging from 1 cm up to 20 cm in greatest dimension [3], but approximately two-thirds of these tumors are found after they have grown over 5 cm [1]. They are well-demarcated from the surrounding liver parenchyma, and their cut surfaces are generally gray-yellow in color with multiple irregular hemorrhagic areas. Necrosis is rare, and prominent central scarring has not been described elsewhere as far as we are aware.

The histologic architecture of hepatic carcinoid can vary among solid, nested, trabecular, and microacinar arrangements [3], as the latter two patterns predominated in this case. The cells themselves are usually small and uniform with round, centrally placed nuclei and granular chromatin. Nucleoli are inconspicuous. Intratumoral hemorrhage can be abundant while the mitotic rate is usually low, both of which characterized our lesion. Necrosis is usually not present; and when it is identified, it is more often focal or punctuate, in stark contrast to the extensive necrotic areas we observed in this case.

Most primary hepatic carcinoids display typical histologic features. Soga, however, reported a group of 19 carcinoids designated as "atypical." We regret that the specific criteria used to designate such cases as atypical are not discussed. The lesion in this case showed few mitoses but revealed mild atypia and exuberant necrosis. Its massive size, sinusoidal infiltration, and intravascular metastases furthermore suggest that this tumor should be described as an atypical variant.

The immunohistochemical characteristics of hepatic carcinoid include stain positivity for the neurosecretory markers chromogranin, synaptophysin, and neuron-specific enolase. Cytokeratin tends to impart a granular perinuclear staining pattern. Markers for gastrin, serotonin, carcinoembryonic antigen, and pancreatic polypeptide are inconsistently positive [1]. Stains for hepatocellular carcinoma (HCC), including Hep-Par1 and AFP, are negative.

The neoplasm in this case mimics several more common hepatic malignancies that may need to be considered in subsequent cases. Grossly, the large irregular central scar brought to mind the possibility of a fibrolamellar variant of heptocellular carcinoma. Then, histologically, the predominantly trabecular architecture coupled with slight nuclear atypia and extensive necrosis was very concerning for HCC. Once the immunohistochemical stains were examined, HCC could be safely ruled out. The neuroendocrine marker positivity prompted us briefly to consider a neuroendocrine carcinoma, but these tumors are typically poorly differentiated and display prominent pleomorphism, nuclear atypia, and a high mitotic index. Because of the presence of scattered acinar-like structures, a mucicarmine stain was performed, the negative result of which ruled out cholangiocarcinoma.

Currently, there do not appear to be any genetic studies conducted on primary hepatic carcinoids, likely a result of this neoplasm's rare occurrence. Multiple studies, however, have discovered cytogenetic and molecular aberrations in gastrointestinal carcinoids from other locations such as the ileum and pancreas [5-8]. As a future endeavor, it may be worthwhile to investigate the possible genetic changes in carcinoids primary to the liver and to compare these changes to those found in other carcinoids.

A complete and thorough autopsy is the only way to prove definitively that a carcinoid is truly primary to the liver, as rectal and ileal carcinoids as small as 1 mm or less have been known to produce large hepatic metastases and comprise the majority of carcinoids identified in the liver [2]. Of course, at this point, an autopsy would be of academic interest only and not for the patient's benefit. In order to safely rule out a non-hepatic primary source, Fenwick et al. provide a diagnostic flow diagram that uses a combination of radiologic imaging studies, somatostatin scan, endoscopy, laparotomy, and regular follow-up appointments [9]. In our case, the multiple radiologic studies that were performed, including a somatostatin scan, coupled with a thorough exploration of the bowel during the surgical resection of the mass led to a strong, although not definitive, clinical diagnosis of primary hepatic carcinoid.

The mainstay of treatment is surgical resection, although some reports have shown the added benefits of systemic chemotherapy and hepatic artery chemoembolus injection [10]. When given as the only form of therapy, chemotherapy drugs such as 5-FU and streptozocin provide a favorable response in only one-third of patients [10].

The overall five-year survival rate for primary hepatic carcinoids is excellent, averaging 92%, while the metastasis rate is 45% [1]. Survival times have ranged from several months up to eighteen years [10]. Of the seven cases of atypical carcinoid followed post-operatively in an article by Soga, all patients were alive well past thirty months [1]. These numbers are small but so far do not indicate a significant difference in survival times between typical and atypical hepatic carcinoids.

At the time of this writing, our patient has reached the eight-month post-operative milestone and is both symptom-free and disease-free. Of course, it will be of great interest as we continue to follow her for survivorship comparisons and tumor behavior. Will these atypical pathologic features predict a worse outcome? Only time may tell.

## Conclusion

Here we have presented the case of giant primary hepatic carcinoid tumor with a number of atypical features including an enormous size, mild cytologic atypia, abundant necrosis, and intravascular metastases. We also describe the lesion's unusual sinusoidal infiltration of the surrounding liver parenchyma, a feature that has not been described as far as we are aware but could potentially serve as a future prognostic finding. As more cases arise, it may be possible to establish a set of guidelines to define atypia in hepatic carcinoids and other gastrointestinal carcinoids, similar to what has been done for their pulmonary counterparts. Thus far, however, based on the current survivorship data, a typical versus atypical diagnosis for these neoplasms may not be necessary other than for academic purposes. Finally, albeit rare, this entity should not be confused with other, more common hepatic lesions such as hepatocellular carcinoma, which carries a significantly worse prognosis and possibly a different treatment regimen.

## Competing interests

The author(s) declare that they have no competing interests.

## Authors' contributions

DL was the primary resident involved in working up this case and co-wrote the majority of the manuscript. LH was the primary medical student involved in the work-up of this case and structured a large part of the Discussion section. AV was the primary resident involved in the radiologic studies for this case and handled the write-up of the radiologic portion of the manuscript. WD was an attending pathologist involved in this case and was involved in structuring the final version of the manuscript. KL was the primary attending pathologist for this case and co-wrote most of the manuscript with DL. All authors read and approved the final manuscript.
